# Translating stem cell research to the clinic: a primer on translational considerations for your first stem cell protocol

**DOI:** 10.1186/s13287-015-0145-7

**Published:** 2015-08-22

**Authors:** Timothy O’Brien, Michael Creane, Anthony J. Windebank, Andre Terzic, Allan B. Dietz

**Affiliations:** Regenerative Medicine Institute, National University of Ireland, Biosciences Building, Galway, Ireland; Centre for Regenerative Medicine, Mayo Clinic, Rochester, 55905 MN USA; Division of Transfusion Medicine, Department of Lab Medicine and Pathology, Mayo Clinic, Rochester, 55905 MN USA

## Abstract

Over the last two decades, a new therapeutic paradigm has emerged which has changed the way debilitating diseases may be treated in the future. Instead of using small-molecule drugs and devices to ameliorate the symptoms of disease, clinicians may harness the therapeutic power of cells to regenerate and cure diseases which currently represent a major unmet medical need. Advancements in the scientific knowledge of stem cell biology, along with highly encouraging preclinical proof-of-concept studies, in the last several years have served as a launch pad for testing such therapeutics in humans with life-threatening diseases. However, translating basic research findings into human therapy has not been straightforward and has presented many scientific, clinical, and regulatory challenges for scientists and clinicians. In this article, we provide a guidance framework for investigators for the design of early-phase clinical studies using stem cell-based therapeutics. Furthermore, important trial parameters and design features which must be considered before regulatory submission of such studies are highlighted.

## Introduction

Stem cell therapy has enormous potential to alleviate human suffering and to provide solutions to conditions with a current unmet medical need. The number of clinical indications for use of these cells and the powerful therapeutic properties have produced a groundswell of interest by physicians around the world to translate scientific discoveries into patient benefit. Used as drugs, stem cells are required to follow the regulatory pathway of pharmaceuticals into the clinic. Stem cells are often used as drugs in investigator-initiated protocols, and so investigators need to be aware of these regulatory pathways from the earliest stage of the translational process. Furthermore, the nature of cells as drugs is more complex, and the translational pathway to development will require special considerations. The purposes of this review are to serve as a primer for physicians who want their laboratory-based discoveries in stem cell therapy to be translated to clinical trials, to encourage investigators to consider the required regulatory steps from the earliest stage of the translational process, and to improve the efficiency of translation of these important discoveries.

## Regulatory agencies as gatekeepers to translational science

No stem cell trial can proceed without review and approval by regulatory authorities. Regulatory agencies and investigators share a similar goal: to bring safe and efficacious novel therapies to patients. These authorities provide critical, independent assessment of a protocol to determine whether the protocol meets the requirements to reduce risk to patients. To maximize the efficiency of translating discoveries into practice, physicians and scientists must understand how regulatory agencies assess new applications. These agencies must assess the current evidence of the potential for safely testing a novel therapy to determine the risk regarding its use in humans. Safe use in humans takes into consideration the drug (its manufacture, purity, and potency), its route of administration, and the potential adverse effects in the environment of the disease to be treated. The inherent paradox in new drug development is the combination of the assessment of drug safety in the context in which it has never been used. This paradox is managed by measuring the strength of the supporting data (preclinical data and related human clinical trials) in relationship to the risk of potential harm (known or unknown) to the patient. It is reasonable (ethically and morally) to allow a greater risk to those patients with few therapeutic options and a poor prognosis. Understanding and mitigating this risk are the responsibilities of the investigator during the application to regulatory agencies, and effectively addressing the risk through proper preclinical studies and identification of appropriate patient populations can catalyze approval of the protocol. For example, sequential patient enrollment whereby a cohort of patients are put on trial and observed for adverse events prior to the next patient cohort enrollment can reduce the overall risk to patients in the protocol. Other risk mitigation strategies include careful preclinical studies that carefully mimic the clinical trial and careful inclusion criteria that describe a patient population with as uniform a prognosis as possible.

## General comments on when it is appropriate to progress to first-in-human studies

There is controversy in the stem cell field concerning the amount of basic science knowledge required before clinical trials should occur [1]. Although it is true that stem cell therapeutic mechanisms are unknown or hypothetical, many drugs currently in use for decades also lack detailed elucidation of the mechanism of action. Investigators should consider the current knowledge concerning the mechanism of action, the alternative treatment options, the severity of the underlying illness, and (if known) the safety profile of the investigative drug. Final criteria for exposing patients to the risks of these new therapies should be a balance between the knowledge (including mechanism of action and safety profile) of the drug (or its bio-equivalent), an evaluation of the potential alternative therapies, and the ability of the investigator to adequately monitor for drug-related adverse events.

## Risk assessment based on clinical protocol

Key to appropriate risk management are the characterization and understanding of the patient prognosis. Protocols should be designed to identify a specific patient population with as few co-morbidities as possible. A narrowly defined patient population must be weighed against patient accrual, but generally the trial should be performed in a well-defined population with a predictable clinical course. Ultimately, the risk of any drug is measured within the patient. Establishment of drug safety in a patient cohort is the foundation of all future trials of the drug. Understanding the pathology for a specific patient population allows accurate attribution of adverse events to the study drug. Allowance of a broad spectrum of patients into safety trials can markedly complicate this attribution. Complex patient populations therefore require more sophisticated preclinical safety data to accompany the application as well as larger trials and sophisticated measures of attribution. As it is ethical for patients with extremely poor prognosis and no alternative acceptable therapies to assume more potential risk in the evaluation of new drugs, a balance must be struck during the identification of the appropriate patient populations. A uniform patient population with predictable prognosis and few alternative treatment options would be ideal. However, patients with extremely advanced disease may not allow the time to evaluate the safety of the drug in the form of drug-mediated disease progression or may experience disease-mediated co-morbidities that prevent gathering firm evidence of safety. Together, the characterization of the patient population and development of a scheme to capture potential adverse events are the first key steps in determining the type of preclinical data required prior to submission.

## Risks associated with drug manufacturing

Stem cells used in the clinic are drugs and therefore must be manufactured as drugs. The manufacture of cell-based medicinal products must be carefully designed and validated to ensure product consistency and traceability. Control and management of manufacturing and quality-control testing are carried out according to Good Manufacturing Practice (GMP) requirements [2]. GMP include document control, standard operating procedures (SOPs), trained personnel, qualified reagents and equipment, and complete provenance of the manufacture of the drug. Prior to their use, drugs must be screened for purity and potency. Purity in stem cells is usually based on phenotype characterization by flow cytometry. Potency testing is used to confirm that the cell-based drug is biologically active and capable of producing the desired biological effect [3]. Potency is usually based on the association of the phenotype characterization with in vitro activity such as immune suppression and cell differentiation [3]. Data demonstrating consistent manufacturing in the patient population are required. The drugs must also be assessed to ensure that no additional risks to the patients (via bacterial contamination or malignant transformation of the product) were introduced during manufacturing. Together, these elements (purity, potency, lack of additional risk factors, etc.) make up the release criteria necessary for any stem cell medicinals to be administered to patients. Ideally, all data supporting the application to the regulatory agency will be generated identically to the product intended to be administered to humans (i.e., cells manufactured using the same SOPs, materials, and cell sources and meeting identical release criteria) [4]. Although this may not be possible for all demonstrations of preclinical efficacy, it should certainly be applied to toxicology studies. This raises the issues of whether toxicology studies should be undertaken using human cells in immunocompromised animals or in a xenogeneic transplant to animals or using animal cells. If cell products are generated in an identical fashion to the clinical product to be used, the former approach will be necessary. Additionally, if minor changes to the process occur after efficacy or toxicology studies are complete and these do not affect the product, an exception may be sought. Finally, unique attributes associated with using cells, such as release criteria, storage requirements, shipping, and shelf life, require special focused attention to assure the investigator and regulatory agencies that patients in a clinical trial will be treated with the same drug.

## Small-molecule drugs versus cell therapeutic

Although in some aspects they are quite similar, first-in-human (FIH) trials using stem cells differ substantially from the typical FIH trials for small-molecule drugs [5]. Unlike stem cells, small-molecule drugs are composed of one active ingredient that works on a single target of action. Drugs often have a stable pharmacokinetic/pharmacodynamic profile in vivo, and results are easier to interpret because of the presence of well-defined reference standards. In contrast to single-modality drugs, stem cell products are complex and can contain multiple active ingredients that work through multiple parallel mechanistic pathways [3]. Furthermore, stem cells are living organisms that can produce responses in a complex multimodal manner depending on the environmental conditions encountered [3]. For example, this is the case with adherent stem cell populations in which it is understood that they exert therapeutic mechanisms via trophic mechanisms. It is understood that these trophic pathways are highly responsive to the microenvironment and are dynamic over time. Therefore, it is not the same dominant mechanistic pathway in which the cells will work every time. It is this inherent property of the cell therapy which makes it very difficult to define reference standards [6] and suitable assays of potency within the field [3].

Many other differences also exist between the two types of products. For example, in contrast to small-molecule drugs, stem cells are a living product and therefore are administered without terminal sterilization. Furthermore, differences in the absorption, distribution, metabolism, and excretion profile of the stem cell product after administration remain unclear. Breakdown/decay of drug concentrations can be monitored in the case of small-molecule drugs as opposed to stem cell products, where unchecked cell proliferation may occur and go unmonitored. As a result, measurements of cell dose may be far less accurate than those for small-molecule drugs. Furthermore, the anatomical site of administration proposed for the delivery of the stem cells may require surgical intervention or the use of novel cell delivery devices to ensure adequate delivery of cells. Such procedures pose further safety risks. If novel cell delivery devices are required, safety tests must be carried out to ensure that the material of the device in contact with the patient does not elicit a harmful biological reaction. Furthermore, the investigator must ensure that the device is sufficient at delivering the stem cell product without damaging the delivery system or the product itself. As stem cells exhibit sensitivities to both chemical and physical stimuli, the investigator must also ensure that correct cell identity is maintained after the product is passed through the device. Another safety concern is the vulnerability of the administration site (brain/spinal cord). Each implantation site will have different degrees of toxicity associated with cell delivery. In some cases, it may be necessary to re-access the implantation site in order for product removal in the event of potential adverse reactions. This will depend on the individual product specifics and disease indication to be treated.

## What constitutes acceptable data to support clinical studies?

The reviewed literature can be used as a resource in collecting data to support clinical trials. Articles that describe other clinical trials with specific details on the safety of the drug can and should be used to support arguments justifying its safe use in humans. It is important to be aware that other trials will likely differ in the dose, route of administration, disease indication, or important characteristics of the drug used. When identifying data in publications used to support the argument for their use in humans, it is important to critically evaluate the differences in the composition, purity, and potency of the drug used. In cell therapy, this can be difficult as the descriptions of the manufacture and evaluation of the drugs can be rather superficial. Data supporting a related but unequal drug will likely be considered irrelevant. However, in situations such as the use of mesenchymal stromal cells, characterization of the cells as per the International Society for Cellular Therapy criteria [7] should allow the use of supporting data from the literature. Another important issue to consider is the type of animal model used to support a regulatory submission. Animal efficacy studies and animal toxicology studies are significantly different in the number included in the study, the doses tested, and biological readouts (histology and non-target organ involvement). Efficacy studies rarely support the safe use of the drug but are used only for the justification for the logical testing of the drug in humans. Animal models of efficacy are justified if the preclinical efficacy studies were meant to represent highly predictive models of efficacy in humans or to provide a rationale for why it is reasonable to progress to human studies. An ideal scenario would be one in which preclinical data are truly predictive but this is rarely if ever the case. It is important to note that, in the US, animal studies of efficacy are required for testing novel drugs in pediatric patients.

It is important that academic clinicians who are engaged in the translation of stem cell research to the clinic be cognizant of the requirements of the translational process from the beginning of the research program. The science required to allow the clinical use of a novel drug is expected to be held to the most rigorous of standards. A typical mistake of new investigators is to assume that scientific data published in peer-reviewed journals automatically qualify as data supporting a clinical trial; often these data do not. Pre-written protocols for every step of the study, data provenance, equipment validation, reagents, animals, and supply provenance (recording all lot and catalogue numbers and expiration dates and methods to ensure that they have been handled or stored properly prior to use), biometric monitoring of animals prior to treatment, animal study randomization, written description of any deviation from the pre-written protocols, and an independent assessment of the data collected should be taken into consideration to produce data in support of the use of any new drug in humans. These requirements are typical of the Good Laboratory Practice (GLP) used in the pharmaceutical industry [8]. GLP is important for laboratories performing preclinical toxicology studies as it ensures that a system is put in place of documentation and SOPs to allow the entire study reconstruction once the final report has been written. Although it is not an absolute requirement for data in support of a clinical trial, every effort should be made to adhere to or approximate GLP principles. Strong self-evaluation of the lab and its practices (and the appropriate changes needed) is absolutely required to save the investigator time and money during the acquisition of the required preclinical data and to prevent potential repeat of the experiments because of failed rigor.

## Toxicology studies

Safety considerations for all drugs should include acute and long-term toxicology (including effects on non-target organs) at drug concentrations beyond those expected to be used in the protocol, specific to the route of injection [4, 9]. Ideally these studies will be in an animal model of the disease but this is not always possible. The optimal safety data are those using published human trial data with a drug that is a bio-equivalent or that has dose and route of delivery similar to those of the drug. The publication should specifically report safety outcomes. If using such data, be aware that significant scrutiny will be placed on these results.

## Unique considerations of preclinical studies relating to cell products

Cell products are guided essentially by the same regulations applied to standard pharmaceuticals. However, cells used as drugs are often culture-derived. Cells in culture are populations derived from a complex starting material that self-purifies during expansion. The culture process must be robust enough to result in a product with consistent purity and stability. The basis for evaluating the rigor of the safety studies in animals stems directly from the data associated with the reproducibility of the purity, stability, and potency of the cells generated by the protocol. Thus, safety data in support of clinical studies should be produced with a cell culture protocol that meets GMP processing with sufficient supportive data to ensure consistency of the production of the drug (cells).

If the actual product cannot be used for preclinical studies, every effort should be made to ensure that the preclinical studies are done with equivalent cells. If published data are used in support of clinical trials, one must prove drug equivalency. Proving equivalency is difficult and complicated by often inadequate description of manufacturing in published reports. Without equivalency, the drugs cannot be assumed to be functionally or toxicologically similar.

In early studies, animal models are often used to evaluate specific safety concerns regarding the drug or route of injection. Animal safety studies should not be confused with efficacy studies. Safety studies must be specifically designed to sufficiently address the question of safety. One complication of toxicology of stem cell products is whether a human-based cell therapy or animal-based one should be used in these studies. If one needs to use the final product, the former approach will be necessary. This may necessitate the use of immunocompromised animals or a xenogeneic approach. There is no absolute guidance on which approach is optimal and this will need to be considered by the investigator and discussed with the regulator.

One of the challenges in the choice of preclinical animal models is the limited nature of the relevance of many of these models to the human situation. Some models may share similar features in anatomical terms (e.g., pig heart), but rarely are the pathology and pathogenesis identical. For example, in translational research of critical limb ischemia, small animal models of acute hindlimb ischemia are relatively poor surrogates for older humans with a history of hypertension, hyperlipidemia, diabetes mellitus, and cigarette smoking for many decades. In addition, dose equivalency is problematic in animals. For pharmaceutical drugs, the dose size is merely adjusted on the basis of animal weight. There is little evidence that the therapeutic value of cells as drugs relates to a correlative increase in dose. Most likely, cells used as drugs meet some threshold of activity with little benefit from additional cells. Ultimately, cell dosage will require empirical evaluation in humans and the trials should reflect this. Finally, the route of injection has implications for the safety of the approach. It is logical that intramuscular injection of 300 million cells has a different safety profile than those cells injected into the carotid artery. Delivery into tissues by using needles, catheters, or adherence to matrices needs to be carefully evaluated (and maybe evaluated for safety) prior to trial initiation.

Finally, it is critical for investigators to realize that in cases of a clinical trial halted because of the safety of the underlying drug, it is likely that all data in support of the clinical trial will be audited. The rigor required by adherence to GLP will provide the assurance that a review of the data, methods, and conclusions will withstand an arduous audit.

## Phase 1 clinical trial considerations

The primary objective of a phase 1 trial is safety assessment, providing information regarding dosage safety and the presence or absence of adverse reactions. Phase 1 or FIH trials can also provide valuable secondary data such as information on issues of feasibility of administration and also on the drug’s biological activity. Such data can be used to design subsequent trials. The following information has been adapted from the US Food and Drug Administration guidelines [10] and will highlight the points that must be taken into consideration by investigators when designing early FIH clinical trials of cellular therapy products.

### Dose exploration

FIH studies can be designed to explore and assess varying dose ranges. Maximum tolerated doses can be explored where the product is being used for life-threatening diseases in which some toxicities are anticipated and can be adequately justified. However, for cases in which minimal toxicity is expected, the dose to be explored is one that can be used to decipher ranges in which the product will produce its maximum biological and therapeutic potential. In stem cell therapy trials, an additional factor for consideration is the limit on dose production with a focus on establishing a safety profile for the dose that is most feasible to produce.

### Feasibility and delivery

Cell therapy products can require state-of-the-art devices and novel procedures in order to maximize cell delivery. FIH trials can be used to discover any technical issues associated with such procedures.

### Efficacy assessment

Although safety is the primary objective, preliminary data on the product’s efficacy can be assessed. Although most FIH trials will not include a sample size great enough to truly assess the product’s activity, suggestions of efficacy as a result of the treatment will provide encouragement to strengthen the scientific rationale to proceed to a phase 2 trial. Caution must be exercised, however, as phase 1 trials will not include controls.

### Choosing a study population

FIH trials are associated with potential risk of unanticipated side effects for the patients. Therefore, the correct patient choice for such trials is very important. Choosing a patient population can be difficult. However, the trial’s objective is to select a patient population in which there is a reasonable balance between potential risks and benefits whilst also accomplishing the scientific objectives of the study.

As with all clinical trials, patient safety is always a major concern and this is specifically true for FIH trials. The possibility of persistent or permanent side effects coupled with invasive procedures for product delivery deems such trials unfavorable for healthy volunteers. The risk-benefit ratio is not optimal for healthy volunteers and therefore the use of healthy volunteers is not acceptable for FIH cell therapy trials.

Patients with severe disease states may be more suitable for FIH investigational cell therapy trials as the risk-benefit ratio may be more acceptable. Despite this, the selection of the correct study population that will provide interpretable data involves several considerations. Patients with a more advanced stage of the disease may tend to experience adverse events not due to the therapy but as a result of the disease progression. Adverse events as such can lead to difficulty in interpreting efficacy and safety data. However, it may be unacceptable to recruit patients with a less severe disease state. If no-option patients are to be included, it is important to ensure that all of their treatment options have been fully explored and evaluated and that such information is recorded carefully. The optimal patient selection criteria for FIH trials would be those with predictable prognosis, no viable therapeutic alternatives, and sufficient time before significant morbidity or mortality occurs.

### Dose selection

Preclinical strategies can be used to generate sufficient information on whether a specific starting dose has an acceptable risk level. However, dose extrapolation using the allometric scaling method may be less precise than for those of small-molecule drugs. Furthermore, pharmacokinetics and pharmacodynamics for cell-based drugs may not be as straightforward to assess and may be difficult to extrapolate from small animals to humans. It is recommended that, if available, previous clinical data produced using cell-based drugs, even if by a different route of administration, be used to help justify a starting dose for the trial.

### Dose frequency

In most early-phase trials, the administration of the treatment is a single, one-off, dose. Cell drugs differ from small-molecules drugs in where they are administered, metabolized in the liver, and then cleared from the body. However, this is not the case for cell-based drugs, as often such products, once administered, have the ability to persist within the body and may have a duration of activity longer than expected. As a consequence, repeated dosing may not be optimal until pertinent information regarding the toxicity and duration of activity of the cells has been obtained.

### Dose escalation

Staggering of drug administration is recommended if no previous human experience has been obtained with the specific dose in question. In the interest of patient safety, staggering of the treatment minimizes the number of patients who are at risk of the unknown side effects of the drugs. Staggering of the treatment is most often between cohorts. For dose escalation studies, treatment groups can be completed sequentially beginning with the lowest dose. Data should be reviewed by the data monitoring and safety board prior to escalation. The choice of staggering interval between subjects should be chosen in such a way that both acute and subacute adverse events can be monitored. Information on the time course in which acute and subacute adverse events may occur can be obtained from preclinical animal data and previous experience in humans if possible. Furthermore, the duration of the product’s biological activity should be considered when choosing the length of the staggering interval.

### Patient-specific products

Cell therapy products are classified as either autologous or allogeneic. Autologous products involve harvest of cells and re-administration to the same individual. In contrast, allogeneic products are obtained from a selected donor who ordinarily will be healthy and multiple doses may be manufactured for receipt by a number of individuals.

Since cell therapies can take a considerable amount of time to manufacture after collection, the patient’s condition must be taken into consideration. Take for example the patient who has satisfied the enrollment criteria at the point of cell collection; however, in the time it has taken to manufacture the drug, the patient’s disease status has worsened such that he or she is no longer eligible to have any further participation within the trial. To account for such circumstances, it is recommended that the trial’s enrollment criteria contain a set of standards to ensure selection of patients who will still be eligible for participation after the manufacturing process is complete. An alternative option is that the patients, at the time of administration, be required to satisfy an independent set of criteria before they can be deemed fit to receive the product.

Another issue that can be encountered upon product manufacture is failure to successfully generate a product that can be used for administration for the recipient. It is important to consider that the patient’s characteristics can influence such issues. For example, the disease and age of the patients may be predictors of a poor cell yield or cell expansion upon ex vivo culture. It can be argued that likelihood of manufacturing success or failure should be addressed in the batch runs included in the investigational medicinal product dossier in the investigational new drug application prior to the trial approval. However, such studies offer high risk with low benefits and little or no incentive for the donors to provide such data. It is optimal to address these manufacturing questions as part of the phase 1 trial and the data gathered from this can be used to design later-phase trials. Furthermore, these data will highlight to the investigator a set of subject selection criteria that are needed to minimize manufacturing failure.

### Safety monitoring and follow-up to mitigate risk to the patients

Safety monitoring in the FIH trial will depend on the anticipated adverse events associated with the specific product. Preclinical toxicology studies should provide sufficient data to help in the choice of safety and monitoring tests that must be carried out to assess both anticipated and unanticipated safety concerns. Common safety tests include general examination and recording of symptoms, blood chemistry, blood hematology, or echocardiography if cardiotoxicity is a concern. Immunology tests may also be required if the product is allogeneic or poses an autoimmunity risk. However, aside from the general safety tests, specific tests and monitoring related to product-specific anticipated events should be considered. Such tests should be carefully chosen once the capabilities of the monitoring tools and analytic methods available at the trial site have been reviewed. In light of this, specific safety and monitoring procedures relevant to the stem cell product should be implemented prior to the trial initiation. For example, immunological assays such as cell and humoral responses should be evaluated if immunogenicity of the product is a safety concern. If suitable assays to assess this have not yet been developed, retention of the baseline and treatment plasma or serum should be considered. This will enable sample evaluation when such assays are made available. It is also suggested that attempts to evaluate product persistence or biological activity be carried out. Such assessments can be made at the region of administration or from the site of the product’s proposed therapeutic activity. This may be possible only if a biopsy can be easily obtained. In addition, protocols can be put in place to require the appropriate post-mortem studies on tissues/organs to assess the persistence or migration of the product should a patient die during the trial [11]. Furthermore, if applicable to the trial site, imaging studies can be used to monitor any ectopic formation or aberrant cell activity.

Stem cell therapies are still in the experimental phase and therefore uncertainties regarding the frequency or severity of adverse events remain. The inclusion of trial-stopping rules into the protocol can enable the investigator to control the number of patients who are put at risk, particularly if safety concerns arise early in the trial. Stopping rules define the number of events or unexpected deaths necessary to put a temporary halt to trial enrollment or dosing. The inclusion of stopping rules does not imply that if such events occur the trial will be terminated but it allows the trial to be temporarily suspended until an adequate assessment of the situation has occurred. This can be beneficial to the trial as the correct assessment can enable the revision of the clinical trial protocol in a manner which benefits the safety and welfare of the patient. For example, revisions of the trial protocol may be made after the assessment to exclude individuals who are more susceptible or at high risk of developing adverse events.

To further reduce the risk for patients enrolled in the trial, suitable follow-up protocols are suggested to be incorporated into the trial design. Preclinical studies, familiarity with the disease, and expertise with the stem cell product will help investigators choose a suitable follow-up time. In the event that the patient fails to receive the product (that is, disease severity worsens and now the patient fails to meet the inclusion criteria), a suitable follow-up protocol must be in place that allows the risk assessment of the cell-harvesting procedure or any subsequent preparation that the patient may have received before the trial. Long-term patient monitoring is integral to the trial design. Long-term follow-up visits are not required to be as detailed as the initial safety assessments provided in the trial. In some instances, brief phone calls to the patient may be sufficient to obtain the required information. Long-term monitoring usually will focus on post-trial patient survival and frequency of adverse events.

## Final statement of working with regulatory agencies

As with all new therapeutic strategies, our information regarding the biological effect of stromal cells is limited and therefore methods to assess safety and efficacy need to be constantly expanded. Adherent stem cell populations such as human mesenchymal stem cells are often portrayed as stem cells that are a well-understood, homogenous population of cells that exhibit predictable properties. Although they are the most extensively studied and characterized cell type, great diversity exists in how investigators have defined and manufactured these cells. Major differences in terms of cell sourcing, product manufacture, and cell surface marker expression exist amongst different laboratories [5]. In addition, differences in the in vitro and in vivo bioactivity of the cells have been reported and these can vary depending on the donor source. With immense speculation surrounding the field and pressure to deliver effective therapies to patients, product quality and consistency are of utmost importance. Identification of parameters important to the cell safety and efficacy is important to ensure quality. Development of assays and screening for stem cell specific markers early in product development will help build our knowledge about the in vitro and in vivo bioactivity of the cell product. The discovery of biological markers that can predict the intended biologic effect which then can be correlated with a beneficial clinical response is essential. Once identified, these parameters can be controlled for in a manner in which the product can be manufactured with a high degree of quality and consistency.

The investment in the development and refinement of new and existing technologies is ongoing in the stem cell field. The development of more advanced preclinical models such as humanized mice and replacement of the use of animal-derived sera in the culture media with safer alternatives such as human platelet lysate is facilitating the development of safer stem cell products.

Regulatory agencies around the world are interested in promoting the safe and effective investigation of novel therapies. Investigators should not consider that they are working alone or in an antagonistic manner on their novel therapies. We strongly recommend that, when planning to apply for FIH stem cell trials, the investigator become familiar with the country-specific process, read and follow all guidance documents, and engage with the agencies early in the development of their process. In addition, the discipline required with FIH trials is worthy of pursuit and involves a close interaction between academic investigators, industry, and regulators. Progress in the therapeutic use of cell-based therapies requires investigators to have the skills to navigate the regulatory environment, develop appropriately designed clinic trials, and consistently manufacture this new class of exciting therapies.

## Abbreviations

FIH: First-in-human
GLP: Good Laboratory Practice
GMP: Good Manufacturing Practice
SOP: Standard operating procedure
